# Metabolic syndrome and population attributable risk among HIV/AIDS patients: comparison between NCEP-ATPIII, IDF and AHA/NHLBI definitions

**DOI:** 10.1186/1742-6405-9-29

**Published:** 2012-10-04

**Authors:** Paulo R Alencastro, Fernando H Wolff, Renato R Oliveira, Maria Letícia R Ikeda, Nêmora T Barcellos, Ajácio B M Brandão, Sandra C Fuchs

**Affiliations:** 1 Hospital Sanatório Partenon, Health Secretariat of State of Rio Grande do Sul, Av. Bento Goncalves, 3722, Porto Alegre, RS, CEP: 90650-001, Brazil; 2 National Institute for Science and Technology for Health Technology Assessment (IATS/CNPq), Hospital de Clínicas de Porto Alegre. R. Ramiro Barcelos 2350, Centro de Pesquisas, Cardiolab-Hipertensão, Porto Alegre, RS, CEP 90035-003, Brazil; 3 Postgraduate Studies Program in Epidemiology, School of Medicine, Universidade Federal do Rio Grande do Sul, R. Ramiro Barcelos 2600, Porto Alegre, RS, CEP 90035-003, Brazil; 4 Postgraduate Studies Program in Cardiology, School of Medicine, Universidade Federal do Rio Grande do Sul, R. Ramiro Barcelos 2600, Porto Alegre, RS, CEP 90035-003, Brazil; 5 Centro de Pesquisa Clínica Hospital de Clínicas de Porto Alegre, Universidade Federal do Rio Grande do Sul, Ramiro Barcellos, 2350, 5° andar 90.035-000, Porto Alegre, RS, Brazil

**Keywords:** Metabolic syndrome, Population attributable risk, HIV/AIDS, NCEP-ATPIII, IDF, AHA/NHLBI, Waist circumference

## Abstract

**Background:**

Metabolic Syndrome (MetS) is based on the same individual components, but has received several amendments to the original definition. In this study, we verified the prevalence of metabolic syndrome according to different criteria, and the impact of each component on the diagnostic.

**Methods:**

This cross-sectional study enrolled HIV infected patients from a HIV/AIDS reference Center in southern Brazil. Metabolic syndrome was identified according to the National Cholesterol Education Program Expert Panel on Detection, Evaluation, and Treatment of High Blood Cholesterol in Adults (NCEP-ATPIII), the International Diabetes Federation (IDF) and the American Heart Association/National Heart, Lung and Blood Institute (AHA/NHLBI) criteria, and using a standardized questionnaire and blood testing.

**Results:**

A sample of 1240, out of 1295, HIV-infected patients was enrolled. Males were on average older, more educated, and had shorter time since the HIV diagnosis. The population attributable risk (PAR) for waist circumference explained 80% of the prevalence among men and women (AHA/NHLBI criteria). Triglycerides had the highest impact on prevalence of metabolic syndrome according to all criteria, independently of age, skin color and HAART use, among men.

**Conclusions:**

In this large sample of HIV infected patients, the overall prevalence of metabolic syndrome, under either classification, was noticeable and the AHA/NHLBI definition accounted for the highest prevalence.

## Background

Metabolic syndrome (MetS) comprises a set of aggregated risk factors including hypertension, central obesity, abnormal fasting glucose, and dyslipidemia which increase the risk of cardiovascular disease [1-4] and type 2 diabetes mellitus [1-4]. Since the third report of the National Cholesterol Education Program Expert Panel on Detection, Evaluation, and Treatment of High Blood Cholesterol in Adults (NCEP-ATPIII) several amendments have been incorporated in a working definition of MetS [5,6]. The International Diabetes Federation (IDF) criteria are based on the same components, but adopted racial/ethnic cutoffs for waist circumference. It have included waist circumference (WC) as a prerequisite for MetS and the treatment for previous conditions as additional criteria of abnormality [4,7].

The revision of NCEP-ATPIII guideline in 2005 [6,8,9] incorporated the treatment for previous disorders (hypertension, diabetes, dyslipidemia) and reduced the cutoff for serum glucose (100 mg/dL) as criteria for abnormality. Recently, a consensus between representatives of the American Heart Association/National Heart, Lung and Blood Institute (AHA/NHLBI) and IDF presented the AHA/NHLBI definition of metabolic syndrome [10]. Central obesity was removed as a prerequisite, so any three risk factors, out of five, make the diagnosis of MetS.

Changes in the thresholds of MetS components account for differences in prevalence rates of this diagnosis, changing also the population attributable risk (PAR) of their components. Prevalence of MetS have been investigated for non-HIV-infected subjects [11,12], but are scarcely reported for the HIV-infected population [13] or compared to the general population [14]. In addition, some components of MetS are particularly vulnerable to the effect of highly active antiretroviral therapy (HAART) [15,16], and it might be time-dependent. This study verified the prevalence of metabolic syndrome according to the NCEP-ATPIII, IDF and the AHA/NHLBI criteria, and investigated the PAR of each component on prevalence rates among HIV-infected adults.

## Material and methods

This cross-sectional study enrolled HIV infected patients, aged 18 to 79 years, from a public health Center for AIDS Care and Treatment in Porto Alegre, southern Brazil. This hospital is one of three reference centers, which provide HIV treatment for patients living in the metropolitan area, other cities of the state or even from other states. A systematic consecutive sample of outpatients, attending to the center at the time of data collection, with confirmed HIV infection, was eligible. Those with mental retardation, restriction of freedom or pregnant women were excluded. The study was approved by the institutional review board of the Hospital de Clínicas de Porto Alegre- Comissão de Ética em Pesquisa, GPPG: 06-243, which is accredited by the Office of Human Research Protections. All participants signed a consent form.

### Studied variables

Metabolic syndrome according to the NCEP-ATPIII [9] was identified for patients who had at least three out of five components: increased waist circumference (WC; men ≥102 cm and women ≥88 cm), triglycerides ≥150 mg/dL or specific treatment, low high-density lipoprotein cholesterol (HDL-C) (men <40 mg/dL, women <50 mg/dL or specific treatment), blood pressure ≥130/≥85 mmHg or anti-hypertensive treatment, and fasting glucose >100 mg/dL, or previous diagnosis of type 2 diabetes mellitus or anti-diabetic treatment. The IDF criteria for MetS [4,7] were based on abnormal WC, in addition to two or more components as those used by the revised NCEP-ATP III [9]. The WC cutoffs vary by racial/ethnic group, but since there are no specific cutoffs for Brazilians, we used those recommended by the IDF for South Asians (≥90 cm, for men, and ≥80 cm, for women). The AHA/NHLBI definition [10] used the same criteria of the revised NCEP-ATP III [9], but the country specific WC cutoff suggested by the IDF [4,7]. For this study, we used those for South Asians.

Potential confounding factors were also studied: gender, age, skin color (self-assessed), years at school, time since the diagnosis of HIV (reported by the patient and confirmed in medical records), and the use of HAART in the 12 months previous to the interview (recorded in medical record).

### Data collection

Patients were interviewed at their routine medical visits by research assistants trained and certified to participate in the study. A standardized questionnaire was used to collect the data, which included questions pertaining to demographic, socioeconomic, and other characteristics, and quality control for data gathering was carried out for a 10% systematic random sample of the interviews. Variables related with HIV infection, use of HAART were obtained at the interview and confirmed with medical records.

Standardized assessments of blood pressure [17,18] were conducted using a validated automatic device (OMRON CP-705) [19], and the average of eight measurements in two office visits was used to diagnose hypertension. Waist circumference was measured with a flexible inelastic tape placed on the midpoint between the lower rib margin and the iliac crest in a perpendicular plane to the long axis of the body, while the subject stood balanced on both feet, approximately 20 cm apart, and with both arms hanging freely [4,20].

A 12 hours fasting glucose and lipid profile were requested for patients who had not been tested in the last three months. Laboratory tests were performed using standardized techniques [21-24].

### Sample size and statistical analysis

The sample size calculation was based on estimated MetS prevalence rates among patients exposed to HAART (25%) and non-exposed (15%) [14], with 80% of power and 0.05 significance level (two-tailed). A sample size of 510 patients would be necessary whether a ratio of 1:1 of non-exposed to exposed to HAART were enrolled, increasing to 741 patients for a ratio of 1:3.

Bivariate analyses were conducted stratified by gender, and statistical significance was assessed by Pearson chi-square test or analysis of variance, using the Statistical Package for the Social Sciences (SPSS Inc., version 16.0 Chicago, Il, USA). The Interactive Risk Attributable Program (IRAP, version 2.2.0, National Cancer Institute, Bethesda, MD, USA) software [25,26] was used to calculate the PAR and corresponding 95% confidence intervals (95%CI) for MetS components after adjustment for other exposures and potential confounders. The PAR allows estimating the proportion of disease burden causally explained by the components of the MetS [27].

## Results

A sample of 1240, out of 1295, HIV-infected patients was enrolled, 15 refused to participate and 40 fulfilled the exclusion criteria. Table 1 shows that participants were aged 38.6 ±10.1 years and mostly were whites. Males were on average older, completed more years at school, and the length of time since the HIV diagnosis was longer than for women. The use of protease inhibitors, on the other side, was more frequent among women.

**Table 1:** Characteristics of the HIV/AIDS patients [N (%) or mean ±SD]

	**Overall**	**Men**	**Women**	**P value**
		**(N= 1240)**	**(N = 628)**	**(N=612)**
Age (years)	38.6 ±10.1	39.5 ±9.6	37.7 ±10.4	0.003
Years at school	7.5 ±4.1	8.1 ±4.2	6.8 ±3.8	<0.001
White skin color	692 (55.8)	380 (60.5)	312 (51.0)	<0.001
Viral load <50 copies/ml	508 (41.6)	268 (43.4)	240 (39.8)	0.2
CD4 (cels/mm^3^)				0.3
<200	181 (14.8)	99 (16.0)	82 (13.5)	
200-350	295 (24.0)	153 (24.7)	142 (23.4)	
>350	751 (61.2)	368 (59.4)	383 (63.1)	
HAART use	815 (65.7)	420 (66.9)	395 (64.5)	0.4
Protease inhibitor use	468 (37.7)	212 (33.8)	257 (42.0)	0.003
Time since HIV diagnosis (years)	4.9 ±4.2	5.2 ±4.6	4.6 ±3.8	0.01

Table 2 shows that the waist circumference (IDF definition), triglycerides, and HDL-C levels were the most prevalent components. All components levels, but fasting glucose, were significantly different among men and women. Abnormal triglycerides were more prevalent among men, and waist circumference (IDF definition) among women. However, there were no statistically significant differences on MetS prevalence based on the three criteria.

**Table 2:** P**revalence of metabolic syndrome and its components according to criteria, by sex [% and (95%CI)]**

	**Overall**	**Men**	**Women**	**P value**
		**(N= 1240)**	**(N = 628)**	**(N=612)**
Abnormal waist circumference ^ǂ^	20.9 (18.6-23.2)	8.1 (6.0-10.2)	34.0 (30.2-37.8)	<0.001
Abnormal waist circumference ^ǂǂ^	46.4 (43.6-49.2)	34.7 (31.0-38.4)	58.3 (54.4-62.2)	<0.001
Blood pressure ≥130/85 mmHg*	28.3 (25.8-30.8)	31.7 (28.1-35.3)	24.8 (21.4-28.2)	0.007
HDL-Cholesterol <40 or <50 mg/dl*	38.8 (36.1-41.5)	33.8 (30.1-37.5)	44.0 (40.1-47.9)	<0.001
Triglycerides ≥150 mg/dl*	35.9 (33.2-38.6)	41.8 (37.9-45.7)	29.9 (26.3-33.5)	<0.001
Fasting glucose ≥100 mg/d*	15.1 (13.1-17.1)	15.1 (12.3-17.9)	15.0 (12.2-17.8)	0.9
Prevalence of metabolic syndrome				
NCEP-ATPIII	17.2 (12.1-22.3)	15.2 (7.9-22.5)	19.2 (12.1-26.3)	0.06
IDF	22.1 (17.2-27.0)	20.7 (13.7-27.7)	23.5 (16,5-30.5)	0.2
AHA/NHLBI	24.7 (19.8-29.5)	24.6 (17.7-31.4)	24.8 (17.9-31.7)	0.9

* Abnormal values or on pharmacological treatment.

^ǂ^Cutoffs by sex: NCEP-ATPIII ≥ 102 cm in men, ≥ 88 cm in women.

^ǂǂ^Cutoffs by sex: IDF and AHA/NHLBI definition ≥ 90 cm in men, ≥ 80 cm in women as the thresholds for South Americans.

**Figure 1:**
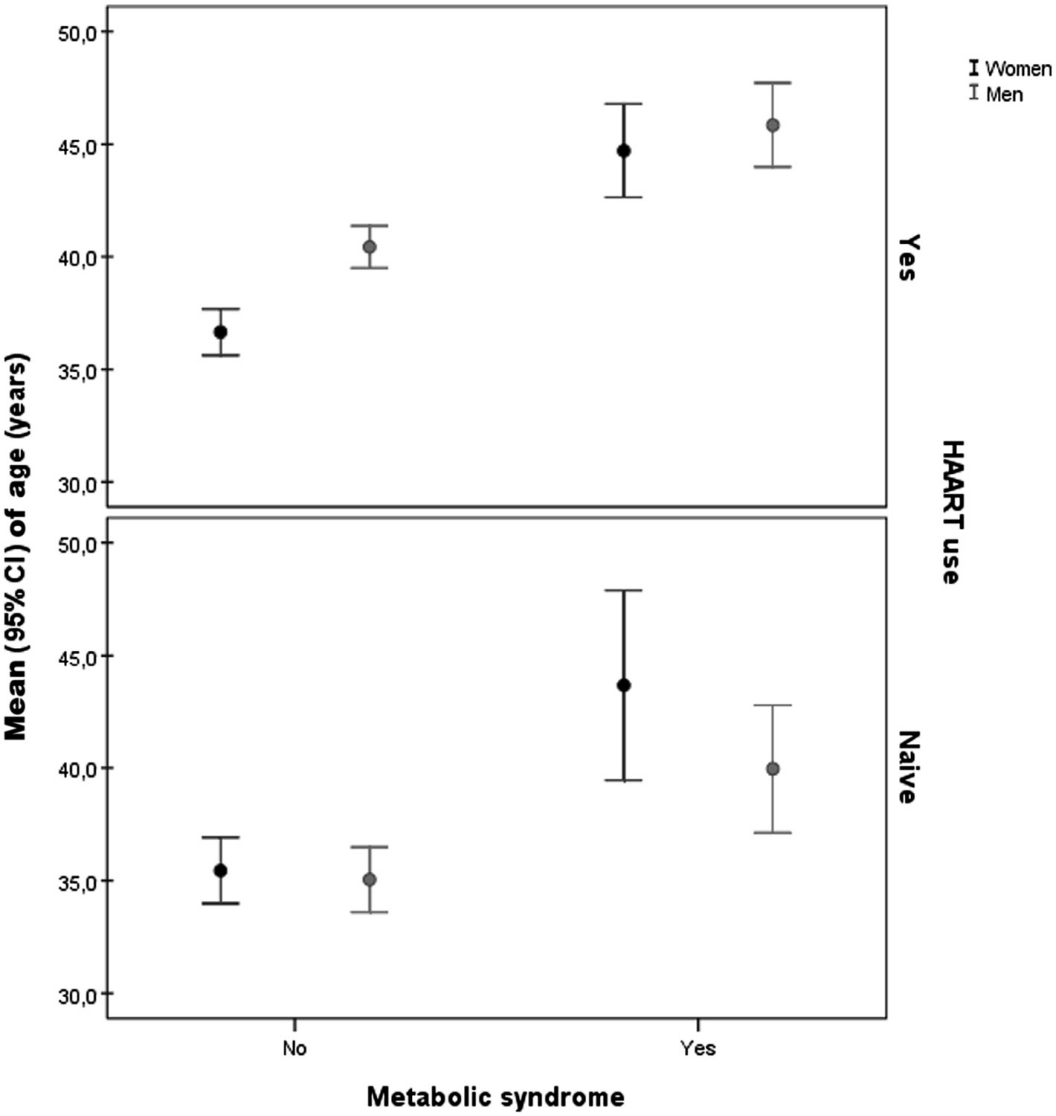
Age distribution of subjects with Metabolic Syndrome according to the AHA/NHLBI (P value <0.001) by sex (P value = 0.001) among HAART users and naïve participants (P values <0.001 and 0.1, respectively).

Figure 1 shows that metabolic syndrome by AHA/NHLBI definition increased with age (P value <0.001) for men and women (P value= 0.001) on HAART use. However, among treatment naïve participants it increased with age (P< 0.001), but it did not vary markedly by sex (P value= 0.1).

Table 3 shows the adjusted PAR for components of the MetS by sex. The PAR for waist circumference explained 80% of the prevalence of AHA/NHLBI definition among men, while triglycerides accounted for the highest impact on prevalence of metabolic syndrome according to all criteria, independently of age, skin color and HAART use.

**Table 3:** **PAR**^**†**^**(95%CI) for metabolic syndrome components according to the criteria, by sex**

	**NCEP-ATPIII**	**IDF**	**AHA/NHLBI**
Men			
Abnormal waist circumference *	33.1 (23.1 - 43.1)	100.0	80.6 (73.3-87.8)
HDL-Cholesterol <40 or <50 mg/dl^ǂ^	65.2 (52.9 - 77.5)	37.9 (25.2 - 50.6)	50.2 (39.6-60.7)
Triglycerides ≥150 mg/dl	88.8 (80.9-96.7)	74.3 (64.7 - 83.9)	79.4 (71.7-87.2)
Fasting glucose ≥100 mg/d	42.6 (31.2 - 53.9)	33.5 (23.9 - 43.2)	37.1 (28.5-45.7)
Blood pressure ≥130/85 mmHg	68.0 (56.5 - 79.6)	55.4 (44.2 - 66.7)	59.2 (49.5-68.9)
Women			
Abnormal waist circumference *	80.4 (72.1-88.7)	100.0	89.8 (82.9-96.7)
HDL-Cholesterol <40 or <50 mg/dl^ǂ^	66.7 (55.8-77.6)	60.7 (50.1-71.2)	63.0 (53.1-72.9)
Triglycerides ≥150 mg/dl	64.1 (53.5-74.7)	61.2 (51.7-70.8)	62.2 (53.0-71.4)
Fasting glucose ≥100 mg/d	44.5 (34.5 - 54.4)	38.9 (30.0-47.9)	40.8 (32.3-49.4)
Blood pressure ≥130/85 mmHg	62.6 (52.5-72.8)	57.4 (47.9-66.9)	58.8 (49.7-67.8)

^†^ PAR: Population Attributable Risk adjusted for age, skin color, and HAART use.

* NCEP-ATPIII (National Cholesterol Education Program-Adult Treatment Panel III) ≥ 102 cm in men, ≥ 88 cm in women; IDF (International Diabetes Federation) and AHA/NHLBI (American Heart Association/National Heart, Lung and Blood Institute) definition ≥ 90 cm in men, ≥ 80 cm in women according the thresholds for South Americans.

For women, waist circumference explained at least 80% of prevalence of MetS according the NCEP-ATPIII or AHA/NHLBI criteria, while for the IDF definition similar impacts were detected for HDL-C, triglycerides, and blood pressure components.

## Discussion

In this large sample of HIV infected patients, the overall prevalence of metabolic syndrome, under either classification, was noticeable. The AHA/NHLBI definition accounted for higher prevalence of MetS than those observed in the NCEP-ATPIII and IDF, which is in accordance with lower cutoff and lacking of the obligatory abnormal waist circumference. The overall prevalence of most MetS components differed considerably among men and women, but the overall prevalence did not vary by sex. Even though the three definitions of MetS were based on the same components, the cutoffs for waist circumference differ markedly on NCEP-ATPIII and IDF, as well as the hierarchy of the central obesity.

This study also estimated the PAR in order to assess separately the contribution of each component on MetS prevalence. This approach assumes that the abnormal MetS components are randomly distributed among the HIV-infected population, but they might be clustered as a consequence of HAART, and, therefore, the PAR could be overestimated. HAART can cause abnormality on lipids and glucose metabolisms [15,16], but these changes could also be caused by the HIV infection [28,29]. In order to minimize the confounding factors, PAR were adjusted for age, skin color, and HAART use.

This study detected lower prevalence rate of MetS, by NCEP-ATPIII definition, than previously described [30-32], which could be attributed to the similar number of men and women, and to the 66% of subjects on HAART, versus the dominance of men and HAART use in those studies. The estimate of MetS based on the IDF definition verified in this study was similar [14] or higher [13] than other studies, which can be partially explained by the high lipodystrophy rate detected among Italian volunteers [33], and the waist circumference cutoffs, which for Brazilians should be the same as those recommended for the South Asians [4,7].

This study detected high prevalence of MetS for men and women with abnormal waist circumference by the IDF and AHA/NHLBI definition. The cutoffs for waist circumference used for South Asians might be excessively low for Brazilians, but they have been previously used in different contexts among non HIV-infected populations [34,35]. The trend for higher prevalence of metabolic syndrome among women might be related to their increased prevalence of central obesity, abnormal HDL-cholesterol, and use of protease inhibitors in comparison with men. Since waist circumference is a mandatory component of the IDF, its impact on MetS prevalence was conceivably 100%.

This study detected lower MetS prevalence by IDF than observed in the general population of Greece [36,37]. Probably, those differences could be attributed to the cutoffs for waist circumference that used ethnic recommendations for abdominal obesity and to lower mean age of HIV-infected patients, which is directly related to abdominal obesity [38,39].

The highest impact of triglycerides and waist circumference suggest that there are consequences associated with the MetS definition adopted to implement any preventive strategy. However, the other components also contributed to the metabolic syndrome burden, and changing their status could result on a substantial reduction on MetS due to triglycerides in males, or waist circumference, in women. The PAR of MetS components might be used to assess the impact of comorbidities, the need for comorbidities treatment, and the HAART toxicity of specific drugs [40]. Interventions to face the burden of metabolic syndrome as well as to determine the health care priority among those who needs medical attention and are at risk for cardiovascular disease are also potential uses for PAR in this area of knowledge.

## Conclusions

In conclusion, the increasing number of antiretroviral agents, longer duration of HAART use, and ageing of the HIV population, might contribute to the growing prevalence of metabolic syndrome and to reduce the life expectancy of HIV-infected patients. This approach of measuring the impact of MetS components on PAR might be useful for comparing the effect of interventions targeting reduction of metabolic syndrome prevalence among HIV-infected populations.

## Abbreviations

MetS: Metabolic syndrome; PAR: Population attributable risk; NCEP-ATPIII: National Cholesterol Education Program Expert Panel on Detection, Evaluation, and Treatment of High Blood Cholesterol in Adults; IDF: International Diabetes Federation; AHA/NHLBI: American Heart Association/National Heart, Lung and Blood Institute; WC: Waist circumference.

## Competing interests

The authors declare that they have no conflict of interest.

## Authors’ contributions

All authors design the study. PA, RO, and MLI collected and analyzed the data. PA, FHW, AB, and NB wrote the manuscript. SF analyzed the data, made substantial contribution to the discussion, and reviewed the final version of the manuscript. All authors read and approved the final manuscript.
